# Would Oscillometry be Able to Solve the Dilemma of Blood Pressure Independent Pulse Wave Velocity – A Novel Approach Based on Long-Term Pulse Wave Analysis?

**DOI:** 10.3389/fphys.2020.579852

**Published:** 2020-10-08

**Authors:** Alexander Reshetnik, Markus Tölle, Kai-Uwe Eckardt, Markus van der Giet

**Affiliations:** Department of Nephrology and Intensive Care Medicine, Campus Benjamin Franklin, Charité – Universitätsmedizin Berlin, Corporate Member of Freie Universität Berlin, Humboldt-Universität zu Berlin, and Berlin Institute of Health, Berlin, Germany

**Keywords:** aortic pulse wave velocity, vascular damage, non-invasive oscillometric measurement, vascular stiffness, arteriosclerosis

## Abstract

The utility of pulse wave velocity (PWV) as a surrogate parameter of arterial vessel damage (AVD) beyond the traditional brachial blood pressure (BP) measurement may be questioned as changes in BP are often accompanied by the corresponding changes in PWV. We sought to establish a new way for BP-independent estimation of AVD with PWV. We retrospectively analyzed data from 507 subjects with at least one available 24 h ambulatory BP- and pulse wave analysis, performed with Mobil-O-Graph (I.E.M., Stolberg, Germany). Individual relationship between eaPWV and central systolic BP (cSBP) was analyzed for every 24 h recording. The analysis revealed linear relation between eaPWV and cSBP in all subjects, which is described by equation eaPWV = a^∗^cSBP + b. We termed “a” as PWVslope and “b” as PWVbaseline. All available demographic parameters and clinical data were correlated with eaPWV, PWVslope and PWVbaseline. 108 subjects had repeated 24 h recordings. Mean age was 60.7 years and 48.7% were female. 92.5% had hypertension, 22.9% were smoker, 20.5% had diabetes mellitus and 29.6% eGFR < 60 ml/min/1,73 m^2^. Direct correlation was observed between age, SBP and eaPWV, while diastolic BP (DBP) and eGFR correlated inversely with eaPWV. PWVbaseline correlated directly with age and inversely with DBP, while PWVslope didn’t correlate with any inputted parameter. Using simple mathematical approach by plotting eaPWV and cSBP values obtained during ABPM, it is possible to visualize unique course of individual PWV related to BP. Using PWVslope and PWVbaseline as novel parameters could be a feasible way to approach BP-independent PWV, though their clinical relevance should be tested in future studies. Our data underline the importance of BP-independent expression of PWV, when we use it as a clinical surrogate parameter for the vascular damage.

## Introduction

Aortic stiffness (AS) is considered to be associated with increased cardiovascular risk. Thus, European Society of Hypertension recommends screening for elevated AS in hypertension (Mancia et al., 2013). Pulse wave velocity (PWV) and in particular carotid-femoral PWV (cfPWV) is an established non-invasive standard to assess AS (Van Bortel et al., 2012). It has been shown to be associated with increased cardiovascular mortality and morbidity independent from established cardiovascular risk factors (Ben-Shlomo et al., 2014). One of the main problems about establishing PWV as an independent surrogate parameter for arterial vessel damage (AVD) is its physiological intrinsic relationship to blood pressure (BP) level. Higher BP results in a stiffer artery and higher PWV, yet without any change in anatomical structure and physiologic properties of the vessel wall. In contrast, AS represents persistent structural changes in arterial vessel walls. It is thus matter of discussion whether increased PWV reflects persistent damage of the arterial wall or is an expression of elevated BP. Establishment of BP-independent PWV would probably better reflect real vessel damage.

Emerging non-invasive oscillometric devices use mathematical approaches and are able to deliver an estimated aortic PWV (eaPWV) based on pulse wave analysis and wave separation analysis, whereby major clinical determinants are age, central systolic BP (cSBP) of the patient and aortic characteristic impedance (Wassertheurer et al., 2008). Validation studies have shown this eaPWV be in good correlation with non-invasively determined cfPWV and invasively determined aortic PWV (Weber et al., 2015; Reshetnik et al., 2017). eaPWV role as a predictor of cardiovascular and all-cause mortality has been shown recently (Sarafidis et al., 2017). Such devices allow easy and quick calculation of eaPWV (Reshetnik et al., 2017), which can be repeated plenty of times under varying BP and patient position. In the present retrospective study we sought to establish new BP-independent PWV using mathematic analysis of the repeated eaPWV measurements during 24 h ambulatory BP and pulse wave monitoring. We hypothesized that establishing of BP-independent PWV would be first step on the way to demonstrate real vascular damage.

## Materials and Methods

All included subjects received long-term (24 h) ambulatory BP and pulse wave monitoring as a part of clinical routine in our Department of Nephrology at Campus Benjamin Franklin, Charité University Berlin. Charité University Berlin review board approved the study (EA4/112/18). No informed consent was required for the study purpose. We screened all available recordings from our database, which comprised time period from August 2012 to February 2018, and included all subjects with at least one representative 24 h recording of peripheral BP, central BP and PWV. All available demographic and clinical data were collected. Smoker status was missing in 50%, exact protein-creatinine ratio in 64% and albumin-creatinine ratio in 65% of study subjects. Other demographic and clinical parameters were completely present in all subjects. We could analyze 648 long-term BP and pulse wave analysis recordings from 507 patients with 43,567 single measurements. Available demographic and clinical data were summarized and analyzed. For the purpose of the study “hypertension” was defined, when the diagnosis “hypertension” has been mentioned in the medical record, subject had antihypertensive medication or the BP level was higher than 130/80 mmHg in the ambulatory BP monitoring. Due to retrospective study design the diagnosis “hyperliproteinemia” and smoking status were based on data from the medical record. All implemented procedures were in accordance with institutional guidelines.

### BP-Monitoring and Pulse Wave Analysis

All recordings were performed with Mobil-O-Graph (I.E.M., Stolberg, Germany) and data analysis was performed with HMS Client Software, Version 5.1. The Mobil-O-Graph is a non-invasive oscillometric device, which combines ambulatory blood pressure monitoring with long-term pulse wave analysis (Supplementary Figure 1). The cuff was applied at the left or right upper arm after the circumference of the arm was measured and appropriate cuff size was chosen (size 1: 24–34 cm or size 2: 32–42 cm). First, brachial SBP and diastolic BP (DBP) were obtained. Thereafter, the cuff was again inflated maintaining the diastolic pressure level for 10 s for assessment of the pulse waveform using high fidelity pressure sensor. The mathematic method for pulse wave analysis in Mobil-O-Graph is based on the algorithm used in ARCSolver (Wassertheurer et al., 2008). Using generalized transfer functions (Fourier analysis and de-compensation into wave harmonics) aortic pressure waveform can be modulated. Central flow curve can be calculated by the means of an adopted, multi-dimensional Windkessel model. The time-lag between pressure and flow curve is generally referred to as “characteristic impedance (Zc),” in which the flow curve follows the pressure curve. Zc, together with the input variables of central systolic and diastolic blood pressure and age allows the device the estimation of aortic PWV (Wassertheurer et al., 2010). Single recordings were done every 20 min during the day (0600–2,200 h) and every 30 min during the night (2,200–0600 h). A 24 h recording with ≥ 80% valid single measurements of SBP, DBP, central BP and PWV was considered representative. For each parameter of a single 24 h recording, mean value of all valid single measurements was obtained and included in the statistical analysis.

### Correlation Between Central SBP and eaPWV

**FIGURE 1:**
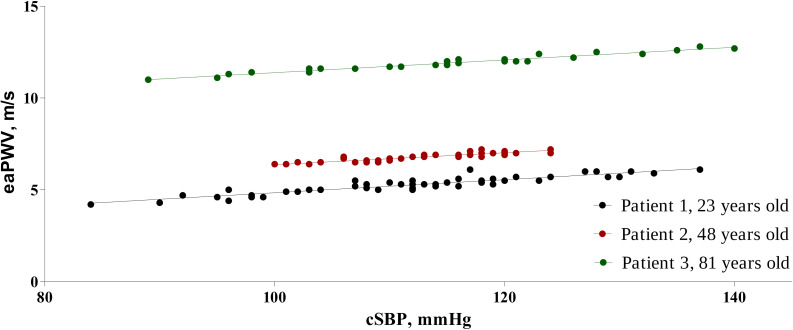
Correlation between central blood pressure and pulse wave velocity in individual patients in three different age ranges; A < 25 years; B- 40–50 years; C > 75 years.

In order to assess the course of eaPWV depending on change in BP, we used scatter plots to visualize a possible correlation. All single measurements of cSBP and corresponding eaPWV from each of the 648 24-h readings were included in separate scatter plots. The relationship between cSBP and eaPWV was linear and could be described with following equation: eaPWV = a^∗^cSBP + b. We termed factor “a” as “PWVslope” and factor “b” as “PWVbaseline.” Figure 1 presents scatter plots with appropriate equations for three individuals from different age ranges.

### Correlation Between Available Demographic and Clinical Data and eaPWV, PWVslope, and PWVbaseline

We tested the impact of each available demographic and clinical parameter on eaPWV, PWVslope, and PWVbaseline using single regression analysis. Those with significant result in single regression analysis were included in multiple regression analysis.

### Repeated Measurements

For a part of the study collective repeated measurements were recorded. One hundred and eight patients had two recordings. Twenty five patients had three recordings. Five subjects had four recordings and one patient had five recordings. Data from at least one follow up recording were compared to the initial measurement, respectively. The change of eaPWV, PWVslope, and PWVbaseline between recordings was assessed and possible factors impacting this change were evaluated.

### Statistics

This study is retrospective. For each continuous variable mean and standard deviation were calculated. For each dichotomous variable number of affected subjects and the ratio related to the whole study collective in per cent were determined. Normal distribution of parameters was proofed with Kolmogorov-Smirnov-Test. In order to compare parameters from repeated measurements paired *T*-Test was used in case of proven normal distribution and Wilcoxon paired test in case of non-normal distribution. A multiple regression approach was used to determine relationship between available parameters. Two-sided *p*-values lower than 0.05 were considered as statistically significant. Statistic analysis was performed with SPSS Statistics 23.0 (IBM, New York, United States).

## Results

Five hundred and seven patients were included in the analysis. Approximately half of the study collective was female (48.7%). The mean age was 60.7 [18–92] years and the mean body-mass index was 27.4 kg/m^2^. 92.5% of patients had established diagnosis of hypertension, 33.7% of hyperlipoproteinemia and 22.9% were smoker. Approximately a fifth had DM (20.5%) and 29.6% have had chronic kidney disease with an estimated glomerular flow rate (eGFR) below 60 ml/min/1,73 m^2^ according to creatinine-based CKD-EPI equation with the mean protein/creatinine-ratio of 363 ± 1,025 mg/g and the mean albumin/creatinine-ratio of 248 ± 792 mg/g. The mean brachial SBP was 133 mmHg and DBP 80 mmHg with the mean heart rate of 69.5 beats/min. The mean cSBP was 121.6 ± 13.4 mmHg and the mean eaPWV was 9.2 ± 2.2 m/s. The mean PWVslope was 0.035 with the range from 0.026 to 0.050 m/s^∗^mmHg and the mean PWVbaseline was 4.9 with the range from −0.21 to 11.2 m/s. Further details regarding demographic and clinical parameters as well as details about medication can be found in Table 1.

**TABLE 1:** Main characteristics and medication of the study collective (*n* = 507).

Sex	
Male, n(%)	260 (51.3)
Female, n(%)	247 (48.7)
Age, years	60.7 ± 16.3
Body mass index, kg/m^2^	27.4 ± 5.3
Hypertension, n (%)	469 (92.5)
Hyperlipoproteinemia, n (%)	171 (33.7)
Smoker, n (%)^∗^	116 (22.9)
Previous stroke, n (%)	26 (5.1)
Coronary heart disease, n (%)	123 (24.3)
Previous myocardial infarction, n (%)	36 (7.1)
Peripheral vascular disease, n (%)	22 (4.3)
Diabetes mellitus, n (%)	104 (20.5)
Chronic kidney disease (eGFR < 60 ml/min/1.73 qm CKD-EPI-Equation), n (%)	150 (29.6)
eGFR, ml/min/1.73 qm	70.4 ± 26.0
Protein/Creatinin-Ratio, mg/g^#^	363 ± 1025
Albumin/Creatinin-Ratio, mg/g^$^	248 ± 792
ABPM brachial systolic blood pressure, mmHg	133.0 ± 14.8
ABPM brachial diastolic blood pressure, mmHg	80.0 ± 10.7
ABPM mean blood pressure, mmHg	101.2 ± 11.3
ABPM pulse pressure, mmHg	52.9 ± 11.3
ABPM heart rate, beats/min	69.5 ± 10.3
ABPM central systolic blood pressure, mmHg	121.6 ± 13.4
ABPM central diastolic blood pressure, mmHg	81.7 ± 10.9
ABPM estimated aortic pulse wave velocity, m/s	9.2 ± 2.2
ABPM PWVslope, m/s*mmHg	0.035 ± 0.003
ABPM PWVbaseline, m/s	4.9 ± 2.2
Medication	
Antiplatelet therapy, n(%)	113 (22.3)
Oral anticoagulation, n(%)	30 (5.9)
Cholesterol reducing therapy, n(%)	180 (35.5)
Statins, n (%)	168 (33.1)
RAAS blocker, n(%)	355 (70)
Calcium-channel blocker, n(%)	275 (54.2)
Aldosterone antagonists, n (%)	37 (7.3)
Thiacid diuretics, n (%)	151 (29.8)
Loop diuretics, n (%)	80 (15.8)
Betareceptor-blocker, n (%)	266 (52.5)
Alphareceptor-blocker, n(%)	63 (12.4)
Central alpha-agonists, n (%)	67 (13.2)
Direct vasodilators, n(%)	15 (3)

*Available in 253 subjects; ^#^available in 181 subjects; ^$^available in 175 subjects.

**FIGURE 2:**
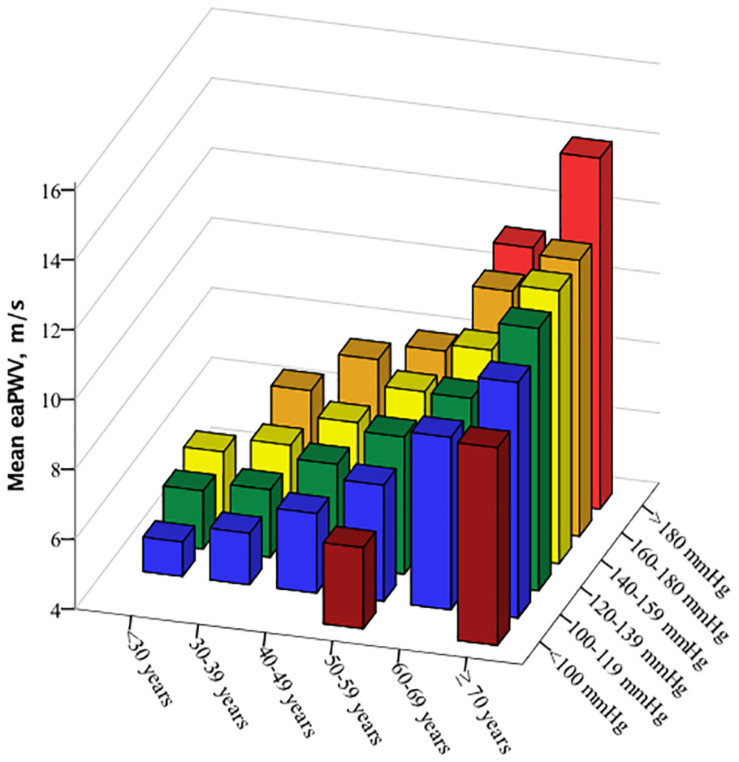
Mean estimated aortic pulse wave velocity (eaPWV) values according to age and blood pressure categories.

**FIGURE 3:**
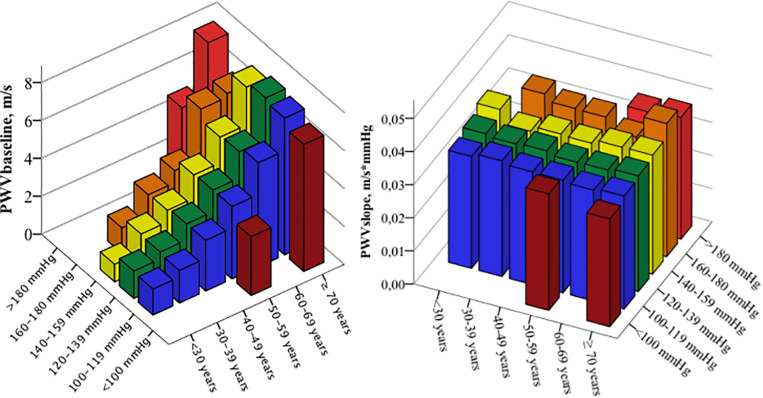
Mean pulse wave velocity baseline (PWVbaseline) values **(right graph)** and PWVslope **(left graph)** according to the age and blood pressure categories.

eaPWV and PWVbaseline increased with rising BP and age (Figures 2, 3). PWVslope increased numerically with increasing age und SBP. This association was without statistical significance (Table 2). eaPWV and PWVbaseline were significantly higher in women compared to men (9.5 ± 2.2 vs. 8.9 ± 2.2, *p* = 0.001; and 5.2 ± 2.2 vs. 4.6 ± 2.1 m/s; *p* < 0.001). No differences in PWVslope were observed between female and male subjects.

**TABLE 2:** Distribution of estimated aortic pulse wave velocity (eaPWV), PWVslope, and PWVbaseline in the study population according to age and blood pressure category.

**Age category, years**	**systolic blood pressure category, mmHg**					
	
	**<100**	**100–119**	**120–139**	**140–159**	**160–180**	**>180**
**eaPWV as mean ± standard deviation, m/s**						
<30		5.0 ± 0.1	5.7 ± 0.8	6.0 ± 0.2		
30–39		5.5 ± 0.2	5.9 ± 0.2	6.4 ± 0.2	7.2*	
40–49		6.3 ± 0.3	6.9 ± 0.4	7.3 ± 0.4	8.3 ± 0.2	
50–59	6.3*	7.3 ± 0.4	7.9 ± 0.4	8.4 ± 0.4	8.8 ± 0.2	
60–69		8.9 ± 0.5	9.2 ± 0.5	9.9 ± 0.6	10.8 ± 0.4	11.2*
≥70	9.7*	10.7 ± 0.9	11.5 ± 1.0	11.8 ± 0.8	11.9 ± 1.3	14.1*
**PWVslope as mean ± standard deviation**						
< 30		0.034 ± 0.002	0.035 ± 0.004	0.037 ± 0.005		
30–39		0.035 ± 0.003	0.035 ± 0.002	0.033 ± 0.004	0.039*	
40–49		0.034 ± 0.003	0.035 ± 0.003	0.034 ± 0.003	0.037 ± 0.002	
50–59	0.035*	0.034 ± 0.001	0.034 ± 0.002	0.035 ± 0.002	0.037 ± 0.003	
60–69		0.033 ± 0.001	0.034 ± 0.003	0.035 ± 0.003	0.034 ± 0.004	0.036*
≥70	0.033*	0.034 ± 0.002	0.035 ± 0.002	0.036 ± 0.004	0.040 ± 0.007	0.037*
**PWVbaseline as mean ± standard deviation, m/s**						
<30		1.4 ± 0.3	1.4 ± 0.9	0.9 ± 0.6		
30–39		1.7 ± 0.2	1.8 ± 0.3	1.9 ± 0.6	1.3*	
40–49		2.7 ± 0.4	2.7 ± 0.4	2.5 ± 0.6	2.5 ± 0.2	
50–59	3.1*	3.8 ± 0.5	3.8 ± 0.5	3.7 ± 0.5	3.1 ± 0.8	
60–69		5.5 ± 0.5	5.2 ± 0.6	5.1 ± 0.5	5.7 ± 1.0	4.9*
≥ 70	6.7*	7.2 ± 0.9	7.4 ± 1.0	7.1 ± 1.0	5.9 ± 0.8	7.7*

*Standard deviation not available.

In the single regression analysis we observed statistically significant correlation between eaPWV and age, SBP, DBP, mean BP, PP, heart rate, sex, DM, hyperlipoproteinemia, eGFR, history of hypertension, coronary artery disease, myocardial infarction, stroke, peripheral arterial disease, medication with ACE-Inhibitor/Angiotensin receptor blocker, calcium channel blocker, thiacids, beta blockers and central alpha-agonists. Multiple regression analysis revealed independent significant influence of age, SBP, DBP, eGFR and history of myocardial infarction on the eaPWV. Increased age and SBP were associated with increasing in eaPWV, while decreased DBP and eGFR were associated with the rise in eaPWV. Study patients with history of myocardial infarction had higher eaPWV than patients without previous myocardial infarction. Using the same demographic and clinical parameters inputted in single and multiple regression analyses we showed independent influence of DBP and age on PWVbaseline (Table 3). In contrast, we observed no statistically significant correlation between any available demographic and clinical parameter and PWVslope, although it increased numerically with increasing age and SBP level.

**TABLE 3:** Significant influence parameters on estimated aortic pulse wave velocity (eaPWV) and PWVbaseline in multiple regression analysis.

	**Dependent parameter: eaPWV**		
**Independent parameter**	**Regression coefficient**	***R*^2^-value**	***p*-value**
Age	0.13	0.86	<0.001
Systolic blood pressure	0.03	0.05	0.007
eGFR	0.002	0.0007	0.02
Myocardial infarction	0.21	0.0006	0.02
**Dependent parameter: PWVbaseline**			
Age	0.12	0.88	<0.001
Diastolic blood pressure	-0.018	0.007	<0.001

One hundred and eight patients have had repeated recordings. The mean between-recording time was 4.5 ± 5.8 months. The mean age of these patients was 60 years and the BMI was 27.8 kg/m^2^. 42% were female. The mean eGFR was 72.1 ml/min/1.73 m^2^ according to creatinine-based CKD-EPI equation with ca. 29% of the patients having chronic kidney disease with an eGFR < 60 ml/min/1.73 m^2^. The mean brachial BP was 134.9/80.1 mmHg with the mean heart rate of 67.3 beats/min. The mean eaPWV was 9.2 m/s with the mean PWVslope of 0.035 and PWVbaseline of 4.9 m/s. Supplementary Table 1 denotes further information about the subgroup of patients with repeated recordings.

eaPWV was adjusted to the SBP level of 120 mmHg. We observed statistically significant difference in eaPWV_120_, while SBP, DBP, PP and heart rate didn’t change significantly between recordings (Table 4). We then investigated change in eaPWV_120_ (delta-eaPWV_120_) across the range of change in SBP, DBP, heart rate and PP as well as change in eaPWV_120_ related to absolute level of initial SBP, DBP, heart rate and eaPWV_120_. Increase in DBP between the measurements was statistically significant associated with decrease in eaPWV_120_ (Spearman *R*^2^ = 0.048, *p* < 0.01). Higher absolute value of initial PP was statistically significant associated with increase in follow-up eaPWV_120_ (Spearman *R*^2^ = 0.038, *p* < 0.05). We did not observe any statistically significant correlation between the change in SBP, heart rate and PP, absolute level of initial SBP, DBP, heart rate, eaPWV120 and change in eaPWV120 between the measurements (Supplementary Figures 2, 3).

**TABLE 4:** Comparison between initial and follow-up recording (*n* = 108).

	**Initial recording**	**Follow up recording**	***p*-value**
Systolic blood pressure, mmHg	134.4 ± 14.3	135.6 ± 15.6	0.35^#^
Diastolic blood pressure, mmHg	80.0 ± 10.7	80.5 ± 11.6	0.59^#^
Pulse pressure, mmHg	54.4 ± 11.3	55.2 ± 11.3	0.34*
Heart rate, beats/min	67.8 ± 9.7	67.4 ± 10.5	0.57^#^
PWV_120_, m/s	8.9 ± 2.1	9.0 ± 2.1	<0.001^#^
PWVslope	0.0347 ± 0.00297	0.03495 ± 0.00324	0.37^#^
PWVbaseline, m/s	4.79 ± 2.11	4.81 ± 2.08	0.56^#^

eaPWV_120_- estimated aortic pulse wave velocity for the systolic blood pressure of 120 mmHg according following equation: PWV_120_ = 120 mmHg*PWVslope + PWVbaseline; ^#^paired t-test; *paired Wilcoxon-test.

## Discussion

In our study analysis of the relationship between eaPWV and cSBP based on multiple single measurements from simultaneous 24 h BP monitoring and pulse wave analysis was essential to describe individual relationship between PWV and BP.

In 2010, a landmark study was published by Arterial Stiffness’ Collaboration, where reference and normal values for PWV (measured as cfPWV) were established. The authors observed a linear relationship between BP and PWV and quadratic relationship between PWV and age (Reference Values for Arterial Stiffness’ Collaboration, 2010). The strength of the study was that all PWV values were measured. However, only few single measurements per patient were obtained and detection of individual relationship between PWV values and corresponding BP level was not possible. Analysis of over 11,000 patients allowed to draw general conclusions about PWV course with change in age and BP in whole study collective. However, individual impact of BP-level on PWV in single patients could not be determined. This point represents a current dilemma in interpreting PWV as an additional cardiovascular risk marker.

In our study, we used a device, which is able to estimate aortic PWV using oscillometric approach. Currently required parameters for the eaPWV calculation are age, measured brachial BP and data from pulse wave analysis (Wassertheurer et al., 2008). Approaching the dilemma of the BP-independent PWV we analyzed individual relationship between eaPWV and SBP based on the data coming from ABPM. Based on our findings the relationship can be described with an individual linear equation. According to determined equation PWVslope could represent individual reaction of PWV to an increase in BP, while PWVbaseline could probably reflect baseline status of the arterial vessels. Though we observed increasing PWVslope values with increasing age and BP, this association was not statistically significant. Additionally, we did not show association between PWVslope and any other clinical parameter. Thus, the clinical relevance of the PWVslope as a separate parameter is still to be proofed. A possible explanation could be a high impact of age in the determination of the eaPWV in the algorithm. Recently, the clinical relevance of the eaPWV beyond the impact of age and SBP has been questioned (Schwartz et al., 2019). Despite the known shot-cuts of the oszillometric PWV estimation this method is valid, feasible and easy to apply in the clinical practice. It is able to capture multiple PWV changes with corresponding changes in BP. Previously to the era of oszillometric PWV measurement multiple PWV recordings were sophisticated and such relationships as obtained in our study could not be established. Based on the mathematical analysis of derived data we were able to reach a “standardization” of the PWV using novel parameters PWVbaseline und PWVslope and in such a way to separate the BP-impact on it. The standardization to a particular BP level is also useful to compare PWV between the patients but also to compare PWV values in the same individual over a time course as we have done in a part of our study collective with available repeated measurements.

To our knowledge, this is the first time serial BP and corresponding PWV changes have been reported. Greve et al. (2016) assessed the performance of estimated PWV, calculated from age, mean arterial pressure, using equations published by Arterial Stiffness’ Collaboration. Though estimated PWV performed well in healthy subjects, it did not add any predictive value in patients with diabetes mellitus or on antihypertensive drugs. As mentioned above, this might be due to high individual variability, which cannot be addressed by using equations coming from another study collective (Greve et al., 2017). Lim et al. (2015) observed intraindividually increasing cfPWV with increasing mean BP in healthy subjects. However, they did not describe individual relationship between cfPWV and BP in their study collective.

The relation between cSBP and eaPWV can readily be translated to the relation between obtained brachial SBP and eaPWV, as we observed well known strong correlation between SBP and cSBP in our data (Pearson correlation coefficient 0.96). We choose cSBP as an independent variable based on the original publication of the method, where authors described cSBP as one of the major determinants needed for the calculation of the eaPWV (Hametner et al., 2013).

We observed well-known association between eaPWV with age and SBP. As in the study published 2010 by Arterial Stiffness’ Collaboration (Reference Values for Arterial Stiffness’ Collaboration, 2010) the correlation between eaPWV and SBP was linear and the correlation between eaPWV and age was better explained by quadratic equation. Furthermore, we saw significant increase in eaPWV with a decreasing kidney function and decreasing DBP. The influence of age, SBP and chronic kidney disease on AS and PWV is well known and could be demonstrated in previous studies (Tolle et al., 2015). The impact of DM on AS (De Angelis et al., 2004) and PWV (Cardoso and Salles, 2016) as its surrogate parameter is also well known. However, we could not show significant independent influence of DM on eaPWV in our collective. One possible explanation could be a relatively low prevalence of DM in our study collective as only circa 20% of patients had DM, which was non-insulin-dependent in the majority of cases. One can speculate that changes in vascular structure might have been only moderate. Furthermore, the impact of BP, age and kidney function had statistically higher impact on eaPWV compared to DM in our analysis. Higher prevalence of isolated systolic hypertension in subjects with DM has been shown previously (Os et al., 2006). Observed increase in eaPWV with lower DBP in our study could thus been interpreted as an indirect link between DM and eaPWV. Supporting this hypothesis, diabetics showed significantly lower DBP compared to non-diabetics in our collective.

Recent data pointed out that changes in heart rate could also contribute to significant changes in PWV (Tan et al., 2016) and adjustment of PWV to a heart rate would also be necessary. We did not see any significant independent effect of heart rate on PWV in our data.

Many studies demonstrated severe differences in PWV between female and male subjects (DuPont et al., 2019). We observed higher eaPWV120 in women. However, we did not show any independent effect of sex on PWV in multiple regression analysis. Additionally, women were significantly older (63.2 ± 15.9 vs. 58 ± 16.4 years) than men in our study collective, which is probably the major reason for higher eaPWV.

Many studies demonstrated significant influence of smoking on AS (Doonan et al., 2010). We did not observe any significant independent effect of smoking on eaPWV. This result is, however, limited as data on smoking status were available in only 50% of the study subjects due to retrospective study design.

Comparison of repeated ABPM readings, which were performed on average 4.5 months apart, revealed statistically significant change in eaPWV120. However, the mean difference of 0.1 m/s is not relevant from the clinical point of view. We observed no clinically relevant impact of any hemodynamic parameter on eaPWV120, no matter whether the amount of difference between initial and follow up recording or absolute value of the initial recording were considered (Supplementary Figures 2, 3), although single variables (e.g., PP) indeed showed statistically significant impact on change in eaPWV120.

Assuming that remodeling of vessel wall is a very slow process, the period of 4.5 months is likely to be too short to reveal a real change in the architecture of the vessel wall explaining why the individual BP-adjusted eaPWV did not change in our study. Thus far, published studies compared initial and follow up PWV-values without individual BP-adjustment. For instance, 2011 published study by Ignace et al. compared cfPWV before and after kidney transplantation (Ignace et al., 2011). Though cfPWV was adjusted to the reduction in mean BP, individual adjustment to the particular level of BP is missing and individual degree of BP-impact on cfPWV cannot be obtained. Whether observed reduction in cfPWV of mean 0.5 m/s 3 months after transplantation in patients still on comparably high level of immunosuppression really reflects improvement in AS is debatable.

## Conclusion

In conclusion, using simple mathematical approach by plotting eaPWV and cSBP values obtained during ABPM, it is possible to visualize unique course of individual PWV related to BP. Using PWVslope and PWVbaseline as novel parameters could be a feasible way to approach BP-independent PWV, though clinical relevance should be tested in future studies. Our data underline the importance of BP-independent expression of PWV, when we use it as a clinical surrogate parameter for the vascular damage.

We acknowledge several limitations of our study: Attributable to the oscillometric method, which we used, obtained PWV-values were not measured but estimated based on pulse wave analysis and utilization of age and central BP. BP-adjustment of eaPWV using obtained individual PWVslope could thus represent the extent of arterial damage. However, the clinical potential of the obtained novel parameters PWVbaseline and PWVslope is not yet established and the next necessary step would be to correlate these parameters to relevant clinical endpoints.

Worth mentioning is the fact that pulse wave analysis is done in the brachial artery, which is a muscular artery, used as a surrogate for PWV in the aorta, which is an elastic vessel. As the anatomy of elastic and muscular arteries is different, they could stiffer in distinct manner (Shirwany and Zou, 2010).

Retrospective design of the study precludes inferences about causal relationships. All available demographic and clinical parameters were included, however, unidentified confounders cannot be ruled out. For instance, no information about the cuff position was available, which is known to be potential influence factor on the PWV. The study population was limited to a specific group of non-severe ill subjects, with a majority having hypertension, mild kidney disease and obesity. Thus, further studies are needed in other populations to confirm and generalize our findings. Low prevalence of diabetes mellitus and advanced kidney disease might have diminished the known effect of these influence factors on PWV. Small part of the subjects with repeated measurements and short follow up period could be a reason for clinically non-relevant change in eaPWV120, obtained in the study.

## Data Availability Statement

The raw data supporting the conclusions of this article will be made available by the authors, without undue reservation.

## Ethics Statement

The studies involving human participants were reviewed and approved by the Ethic Review Board Charité University Berlin. Written informed consent for participation was not required for this study in accordance with the national legislation and the institutional requirements.

## Author Contributions

AR and MG: conceptualization. AR: methodology, formal analysis, writing—original draft preparation. K-UE and MG: writing—review and editing. MG: project administration. All authors contributed to the article and approved the submitted version.

## Conflict of Interest

The authors declare that the research was conducted in the absence of any commercial or financial relationships that could be construed as a potential conflict of interest.
