# Supplementary figures and images for: Would Oscillometry be Able to Solve the Dilemma of Blood Pressure Independent Pulse Wave Velocity – A Novel Approach Based on Long-Term Pulse Wave Analysis?
Source: Front Physiol. 2020 Oct 8;11:579852. doi: 10.3389/fphys.2020.579852 (PMC7579143; doi:10.3389/fphys.2020.579852)

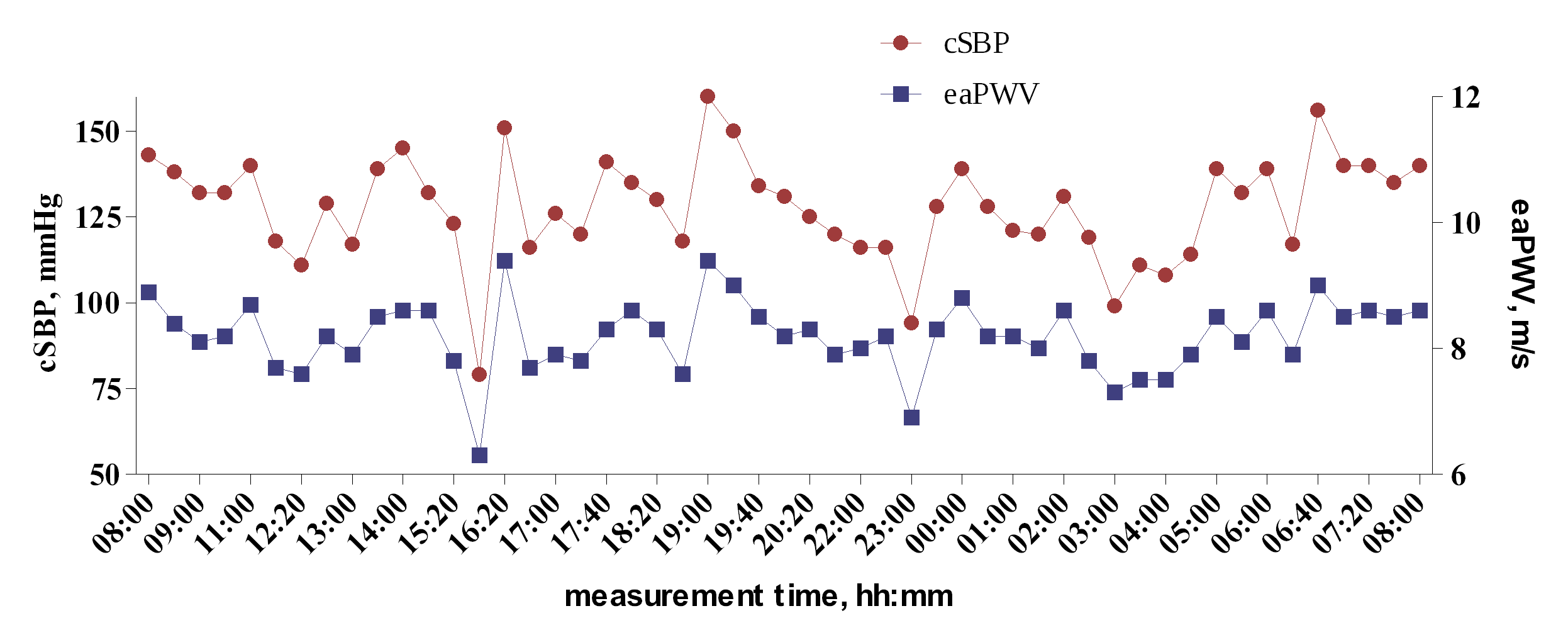

Supplement: Supplementary file 1 [file Image_1.TIF]

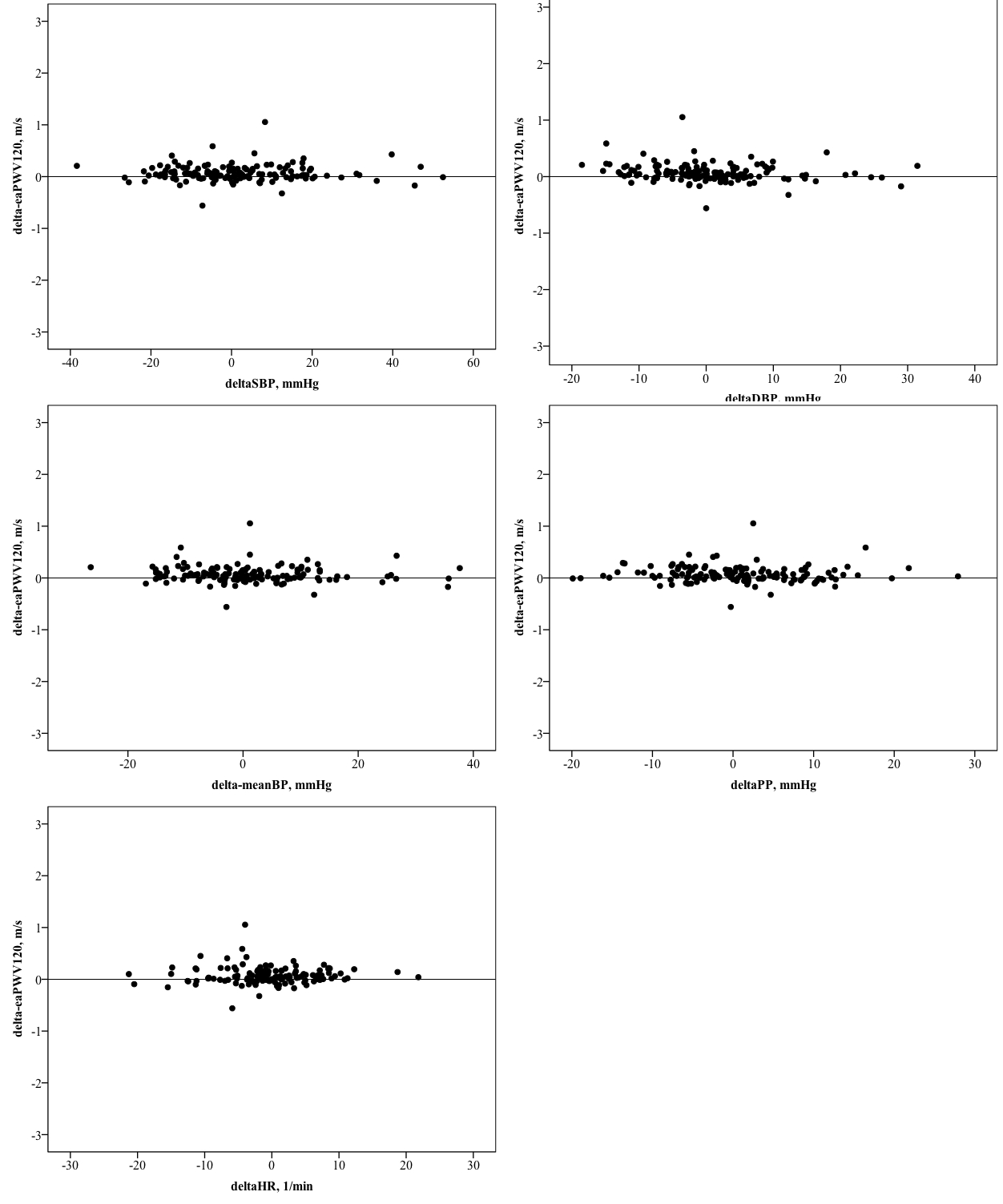

Supplement: Supplementary file 2 [file Image_2.JPEG]

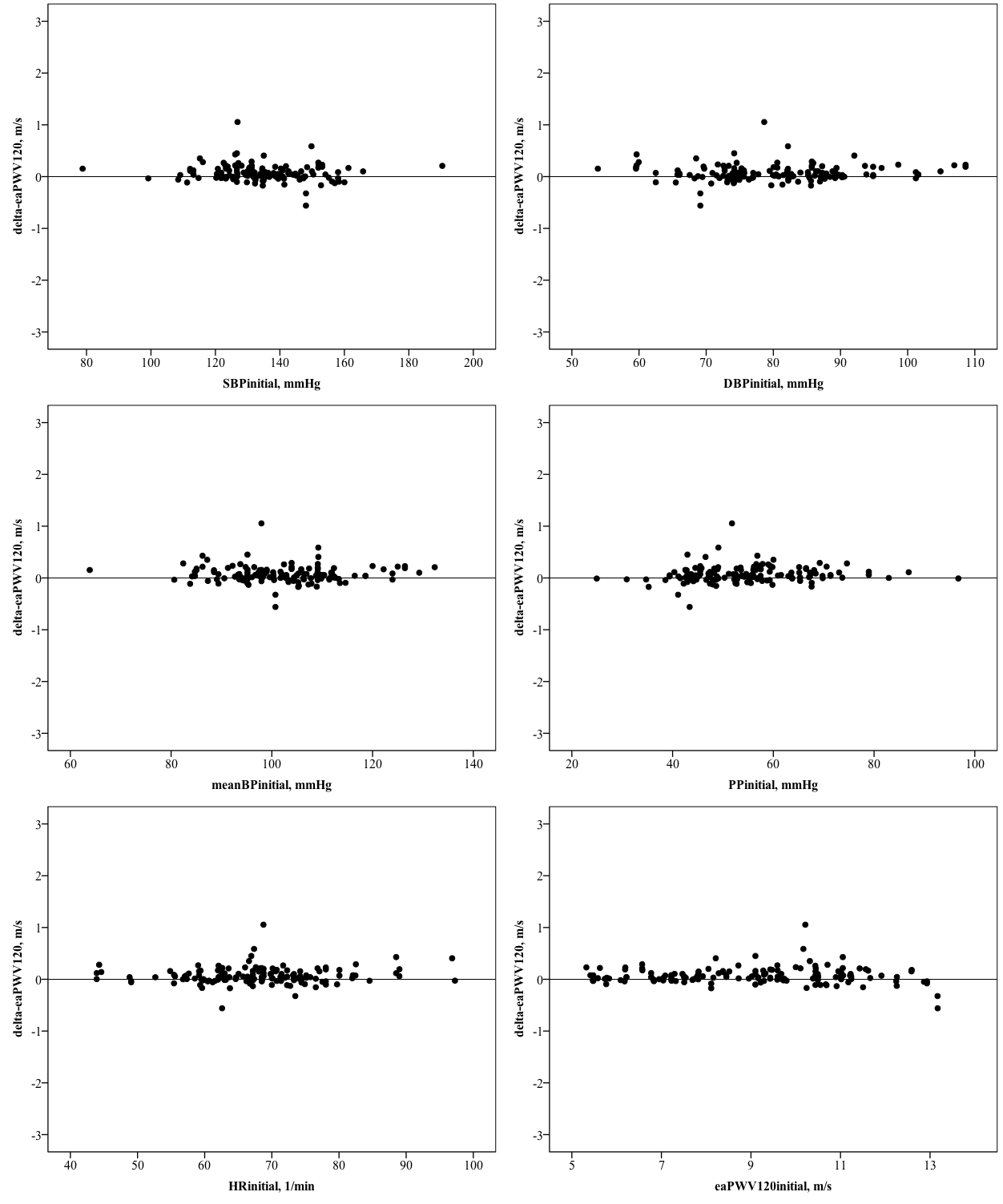

Supplement: Supplementary file 3 [file Image_3.TIF]
