# Supplementary material for: Would Oscillometry be Able to Solve the Dilemma of Blood Pressure Independent Pulse Wave Velocity – A Novel Approach Based on Long-Term Pulse Wave Analysis?
Source: Front Physiol. 2020 Oct 8;11:579852. doi: 10.3389/fphys.2020.579852 (PMC7579143; doi:10.3389/fphys.2020.579852)
Supplement: Supplementary file 4 [file Data_Sheet_1.docx]

# Supplement

Figure 1 shows the course of recorded central systolic blood pressure and estimated aortic pulse wave velocity during the ambulatory monitoring.

Figure 2: Change in estimated aortic pulse wave velocity adjusted to systolic blood pressure 120 mmHg (eaPWV120) related to change in systolic (SBP) and diastolic blood pressure (DBP), heart rate (HR), mean brachial blood pressure (meanBP) and pulse pressure (PP)

Figure 3: Change in estimated aortic pulse wave velocity adjusted to systolic blood pressure 120 mmHg (eaPWV120) related to absolute level of initial systolic (SBP), diastolic blood pressure (DBP), mean brachial blood pressure (meanBP), heart rate (HR) and pulse pressure (PP) and eaPWV120.

Table 1: Main characteristics and medication of the subgroup with repeated measurements (n=108)

| Sex  male, n(%)  female, n(%) | 63 (58.3)  45 (41.7) |
| --- | --- |
| Age, years | 60.0±15.7 |
| Body mass index, kg/m^2^ | 27.8±4.7 |
| Hypertension, n (%) | 106 (98.1) |
| Hyperlipoproteinemia, n (%) | 42 (38.9) |
| Smoker, n (%) | 13 (12) |
| Previous stroke, n (%) | 4 (3.7) |
| Coronary heart disease, n (%) | 22 (20.4) |
| Previous myocardial infarction, n (%) | 4 (3.7) |
| Peripheral vascular disease, n (%) | 6 (5.6) |
| Diabetes mellitus, n (%) | 29 (26.9) |
| Chronic kidney disease (eGFR<60 ml/min/1.73 qm CKD-EPI-Equation), n (%)  eGFR, ml/min/1.73 qm  Protein/Creatinin-Ratio, mg/g  Albumin/Creatinin-Ratio, mg/g | 31 (28.7)  72.1±24.3  298.8±524.7  215.2±466.4 |
| Brachial systolic blood pressure, mmHg | 134.9±15.0 |
| Brachial diastolic blood pressure, mmHg | 80.1±10.2 |
| Pulse pressure, mmHg | 54.8±11.7 |
| Heart rate, beats/min | 67.3±9.6 |
| Central systolic blood pressure, mmHg | 122.8±13.2 |
| Estimated aortic pulse wave velocity, m/s | 9.2±2.1 |
| PWVslope | 0.035±0.0033 |
| PWVbaseline, m/s | 4.9±2.1 |
| Medication  Antiplatelet therapy, n(%)  ……...oral anticoagulation, n(%)  ...........cholesterol reducing therapy, n(%)  …….. Statins, n (%)  ……. RAAS blocker, n(%)  Calcium-channel blocker, n(%)  Aldosterone antagonists, n (%)  Thiacid diuretics, n (%)  Loop diuretics, n (%)  Betareceptor-blocker, n (%)  Alphareceptor-blocker, n(%)  Central alpha-agonists, n (%)  Direct vasodilators, n(%) | 18 (16.7)  6 (5.6)  40 (37.1)  37 (34.3)  86 (79.6)  75 (69.4)  16 (14.8)  42 (38.9)  15 (13.9)  69 (63.9)  19 (17.6)  20 (18.5)  5 (4.6) |

RAAS – renin-angiotensin-aldosterone-system
